# New York State Medical Association

**Published:** 1891-06

**Authors:** William H. Thornton

**Affiliations:** Secretary


### NEW YORK STATE MEDICAL ASSOCIATION.—FOURTH DISTRICT BRANCH.

Reported by WILLIAM H. THORNTON, M. D., Secretary.

THE seventh annual meeting was held at the Genesee Valley Club, Rochester, May 12, 1891.

The meeting was called to order at 11 o'clock A. M. by the President, Dr. R. J. MENZIE, of Caledonia.

The minutes of the previous meeting were read and approved.

THE PRESIDENT stated that his manuscript had inadvertently been left at home, hence the President's annual address was omitted.

Upon motion of the Secretary it was voted that a committee be appointed of one member from each county, excepting Erie County which should have two, who should have charge of the scientific programme for the next meeting.

Dr. R. M. MOORE reported for the Committee of Arrangements that a lunch would be served in the adjoining room at the close of the morning session.

Dr. M. W. TOWNSEND announced the death of Dr. Wm. B. Sprague since the last meeting of the Branch. (See p. 570, April number, of the JOURNAL, for obituary notice of Dr. Sprague.)

The following Nominating Committee was appointed: Dr. N. G. Richmond, Chautauqua; Dr. H. D. Ingraham, Erie; Dr. F. L. Stone, Genesee; Dr. F. H. Moyer, Livingston; Dr. J. D. Dunning, Monroe; Dr. J. H. Taylor, Orleans; Dr. J. Dunn, Steuben; Dr. Darwin Colvin, Wayne; Dr. A. G. Ellinwood, Wyoming.

The first paper presented to the Association was entitled Ectopic Gestation, by Dr. M. W. Townsend, of Genesee County. Discussed by Drs. Ingraham and Richmond.

A paper on Noma following Measles was presented by Dr. D. J. Chittenden, of Steuben County. Discussed by Drs. Hubbell and Ingraham, and the author.

A paper on The Purse String or Tobacco Pouch Operation for Cystocele, with Report of Cases, was presented by Dr. H. D. Ingraham, of Erie County.

The Nominating Committee reported in favor of the election of the following members of the Executive Committee for the ensuing year: Dr. Jas. A. Stevenson, Allegany; Dr. S. J. Mudge, Cattaraugus; Dr. N. G. Richmond, Chautauqua; Dr. H. D. Ingraham, Erie; Dr. F. H. Moyer, Livingston; Dr. M. W. Townsend, Genesee; Dr. R. M. Moore, Monroe; Dr. S. T. Clark, Niagara; Dr. F. D. Vanderhoff, Ontario; Dr. John H. Taylor, Orleans; Dr. Jeremiah Dunn, Steuben; Dr. A. G. Ellinwood, Wyoming; Dr. Wm. Oliver, Yates; Dr. Darwin Colvin, Wayne.

Upon motion of the Secretary the report of the Nominating Committee was received and adopted.

The meeting was then adjourned until 1.45 P. M., and the members partook of a luncheon provided by Drs. E. M. Moore, Jr., and R. M. Moore.

The afternoon session was called to order by the President at 2 o'clock P. M.

The following papers were presented:

Affections of Sight from Injuries, by Dr. Alvin A. Hubbell, of Erie. Discussed by Drs. Rider and Goler, of Rochester.

A New Mode of Making Extension and Counter-Extension in the Application of the Plaster Jacket for Curvature of the Spine, by Dr. Jeremiah Dunn, of Steuben. Discussed by Dr. E. M. Moore, Jr., of Rochester.

Vesico-Urethral Fissure in Women, by Dr. G. W. Goler, of Monroe. Discussed by Dr. E. M. Moore, Jr., of Rochester.

The Treasurer presented his annual report showing receipts of $57.73; expenses, $17.10; balance, $40.63. He recommended that there be no assessment levied for this year. The report of the Treasurer was adopted.

THE PRESIDENT appointed the following Committee on Scientific Programme for the ensuing year: Allegany County, Dr. A. W. Cottrell; Cattaraugus County, Dr. V. A. Ellsworth; Chautauqua County, Dr. T. D. Strong; Erie County, Drs. A. A. Hubbell and Jas. W. Putnam; Genesee County, Dr. F. L. Stone; Livingston County, R. J. Menzie; Monroe County, Dr. G. W. Goler; Niagara County, Dr. Wm. Q. Huggins; Ontario County, Dr. Jas. H. Allen; Orleans County, Dr. H. C. Tompkins; Steuben County, Dr. D. J. Chittenden; Wayne County, Dr. J. N. Arnold; Wyoming County, Dr. Z. J. Lusk; Yates County, Dr. William Oliver.

THE PRESIDENT congratulated the meeting upon the interesting papers and discussions which had been presented, and declared the meeting adjourned.

